# Supplementary material for: Analysis of Polar Lipids in Hemp (Cannabis sativa L.) By-Products by Ultra-High Performance Liquid Chromatography and High-Resolution Mass Spectrometry
Source: Molecules. 2022 Sep 9;27(18):5856. doi: 10.3390/molecules27185856 (PMC9503808; doi:10.3390/molecules27185856)
Supplement: Supplementary file 1 [file molecules-27-05856-s001.zip › molecules-1883902-supplementary.pdf]

**SUPPLEMENTARY DOCUMENT**

# Analysis of Polar Lipids in Hemp (*Cannabis sativa* L.) By-Products by Ultra-High Performance Liquid Chromatography and High-Resolution Mass Spectrometry

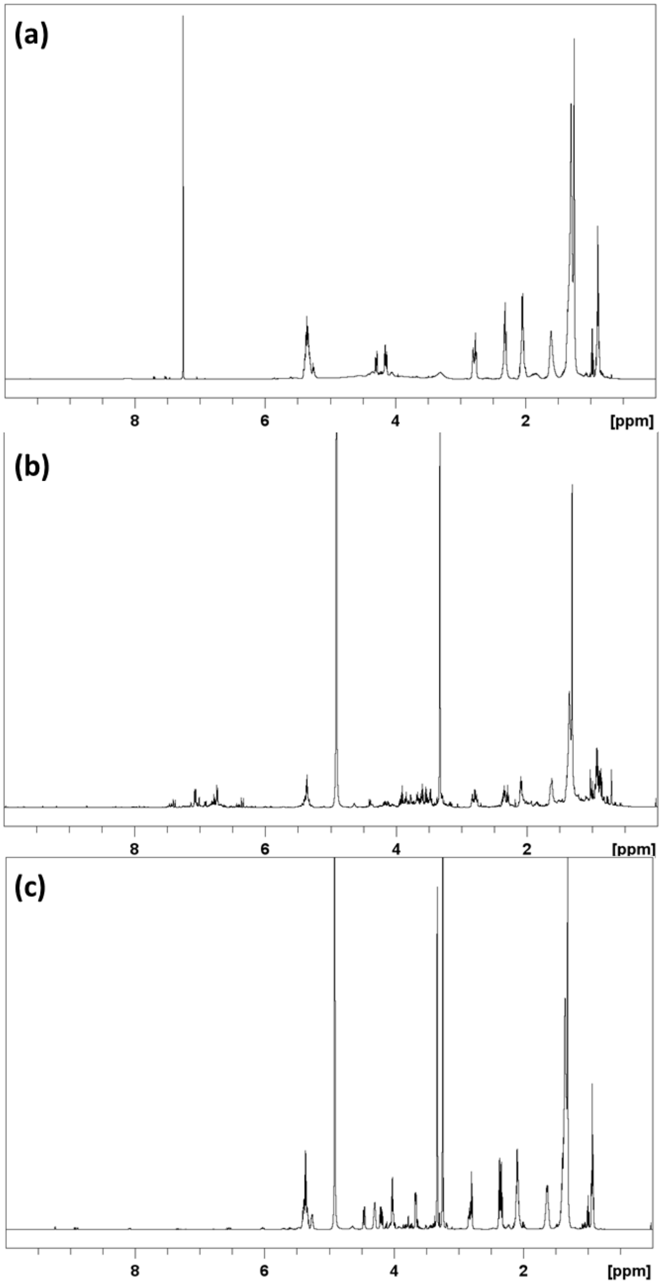


**Figure S1.** $^{1}H$ NMR spectrum of hemp cake SPE fractions chloroform (a) acetone (b) and MeOH (c).

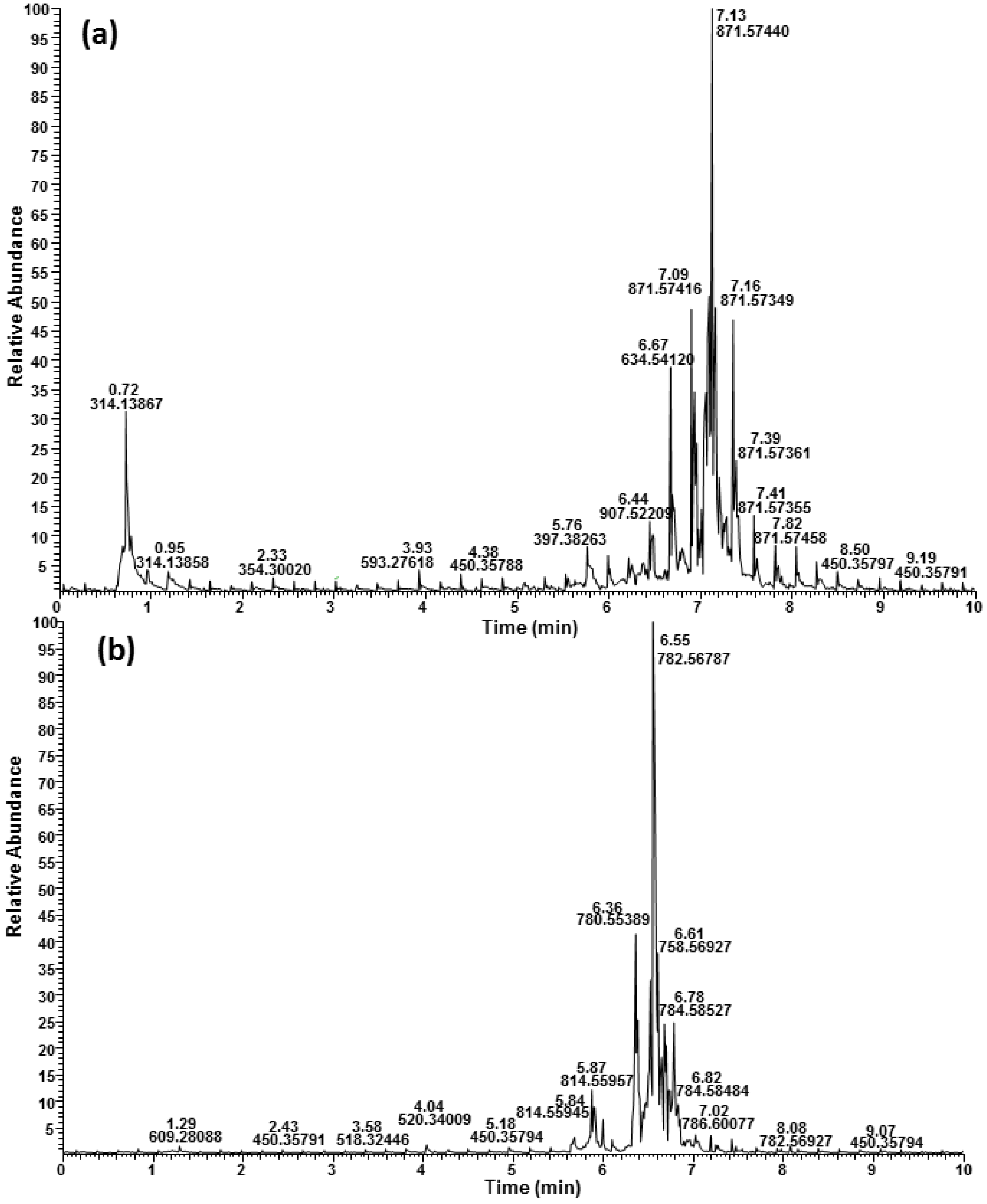


**Figure S2.** TICs of hemp cake SPE fractions acetone (a) and MeOH (b) in positive mode.

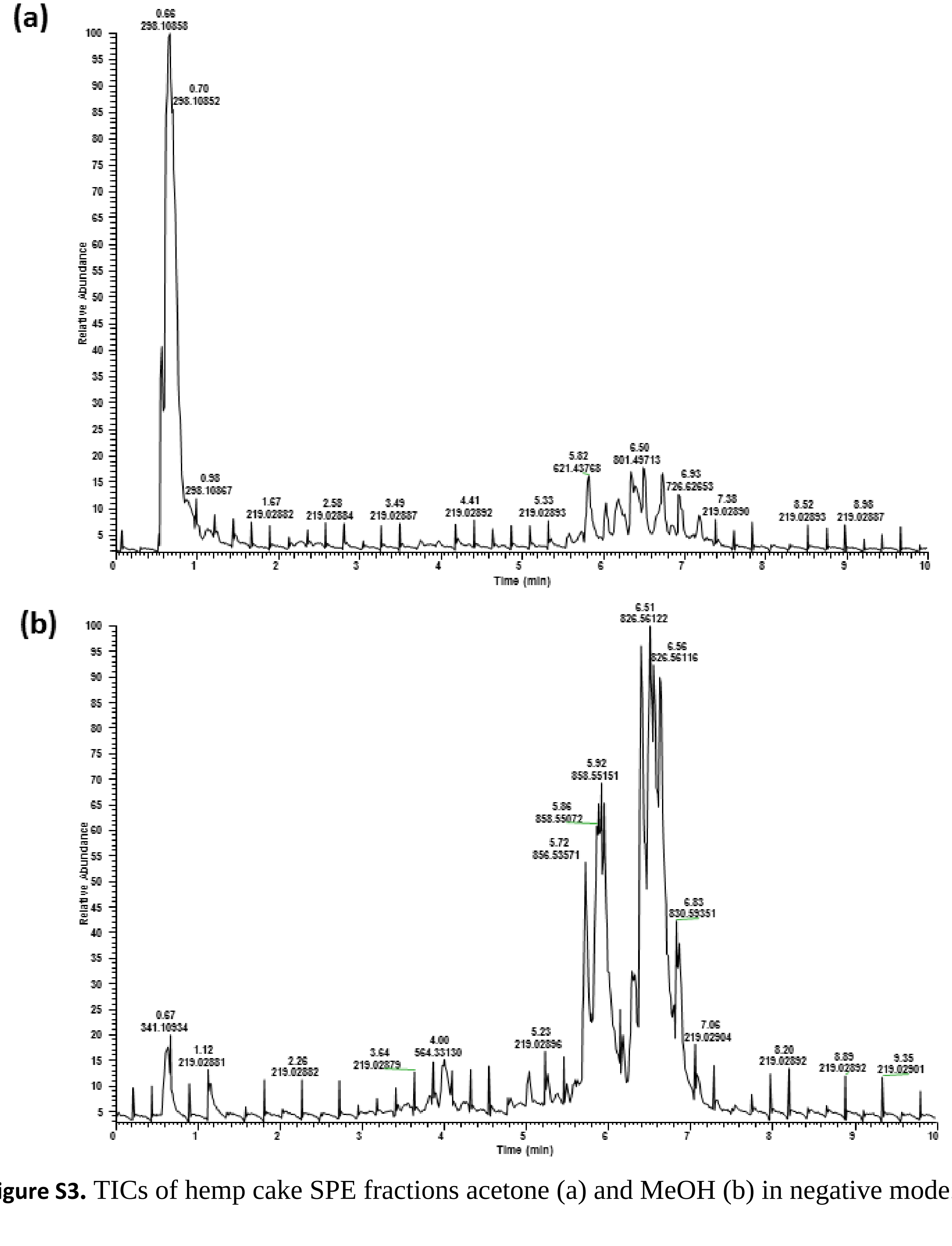


**Figure S3.** TICs of hemp cake SPE fractions acetone (a) and MeOH (b) in negative mode.

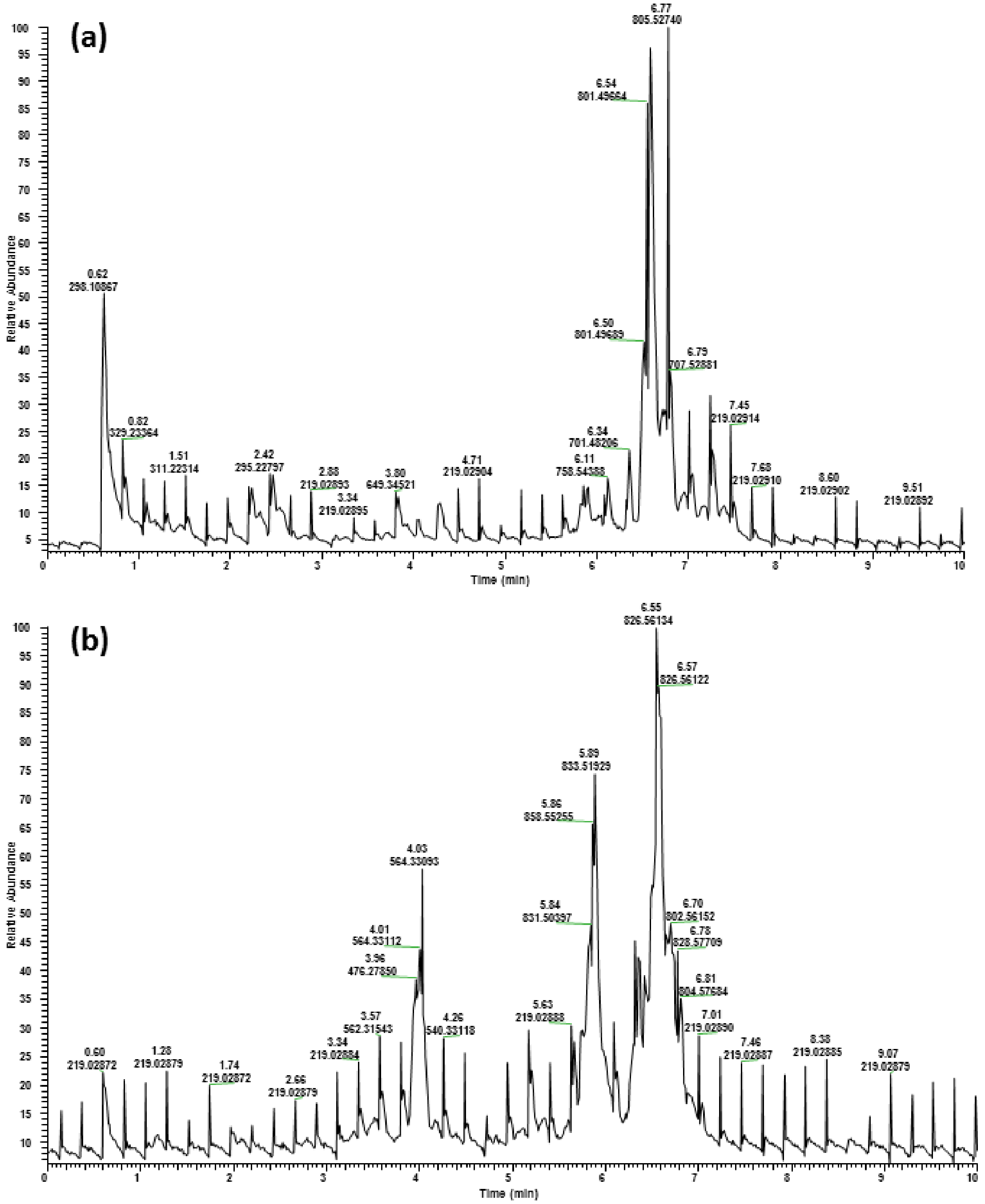


**Figure S4.** TICs of hemp seed hulls SPE fractions acetone (a) and MeOH (b) in negative mode.

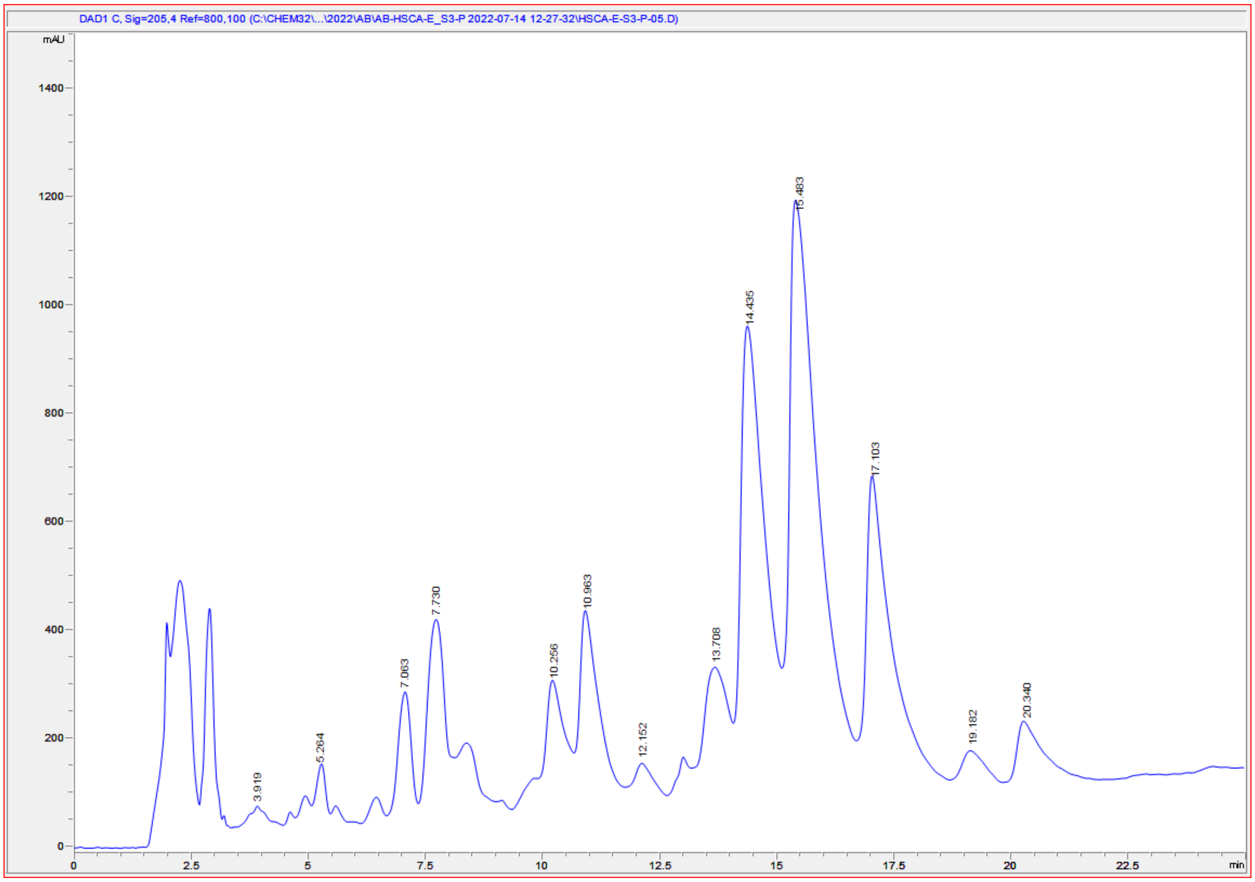


**Figure S5.** HPLC chromatogram of MeOH fraction of hemp cake.

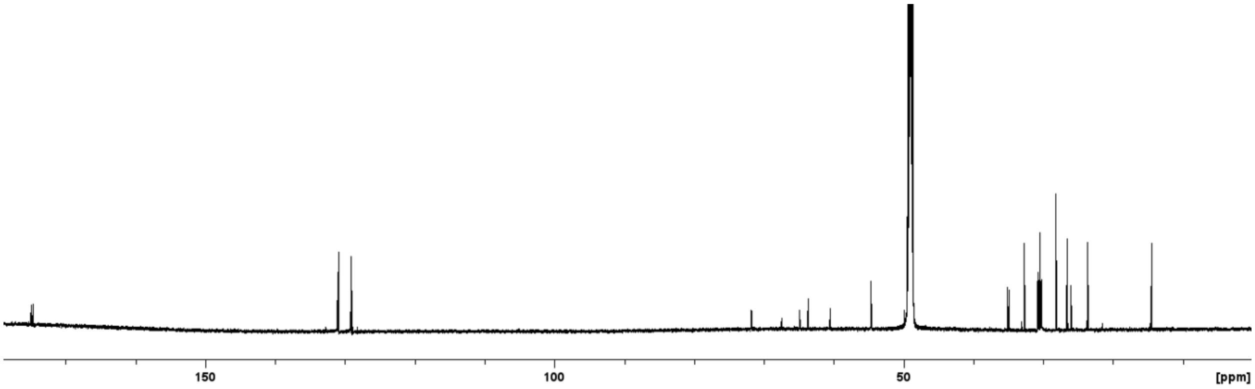


**Figure S6.** The $^{13}$C NMR spectrum of 1,2-di-linoleoylphosphatidylcholine (PC 18:2/18:2) measured in $CD_3OD$.

**(a)**

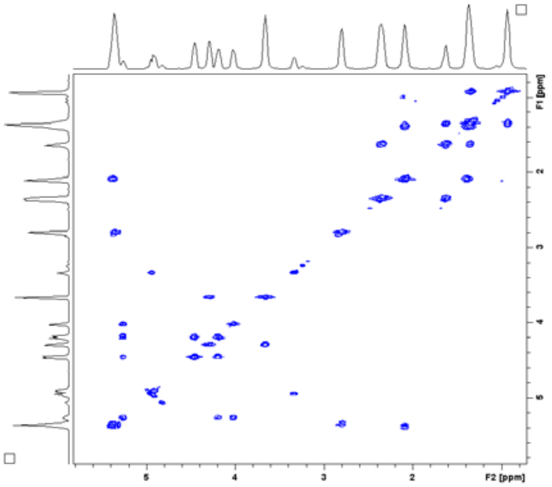


**(b)**

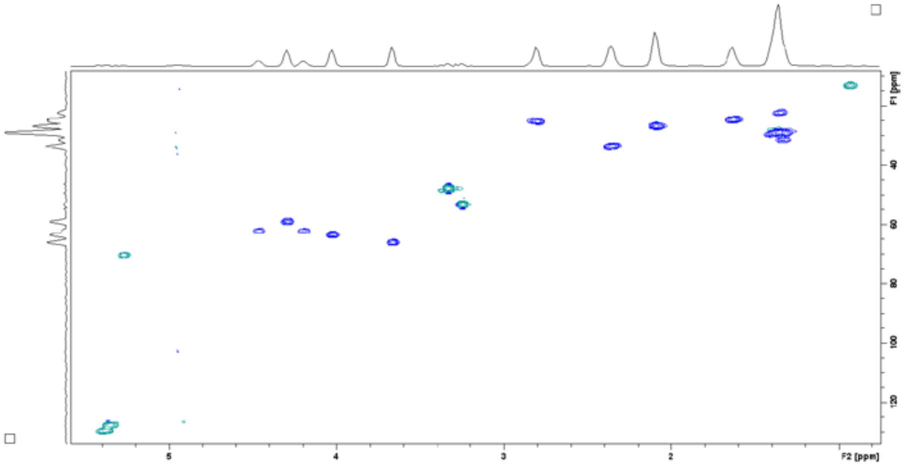


**(c)**

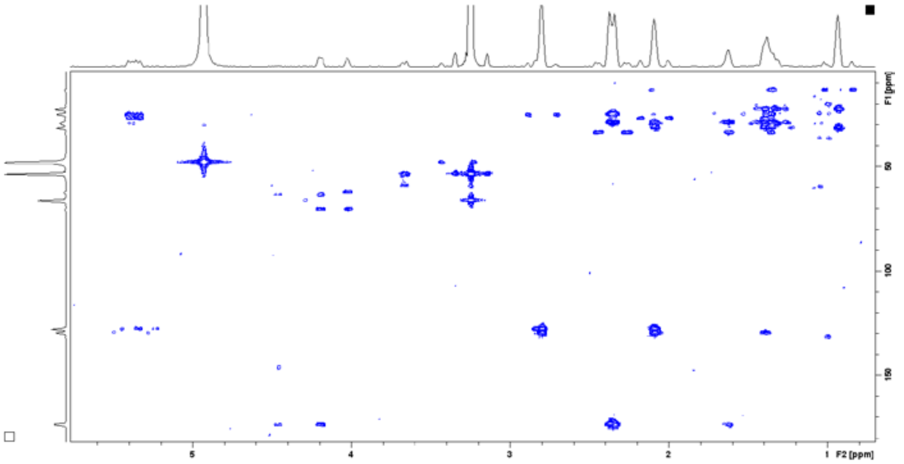


**Figure S7.** 2D-NMR spectra of 1,2-di-linoleoylphosphatidylcholine COSY (a) HSQC (b) and HMBC (c) measured in $CD_3OD$.

**(a)**

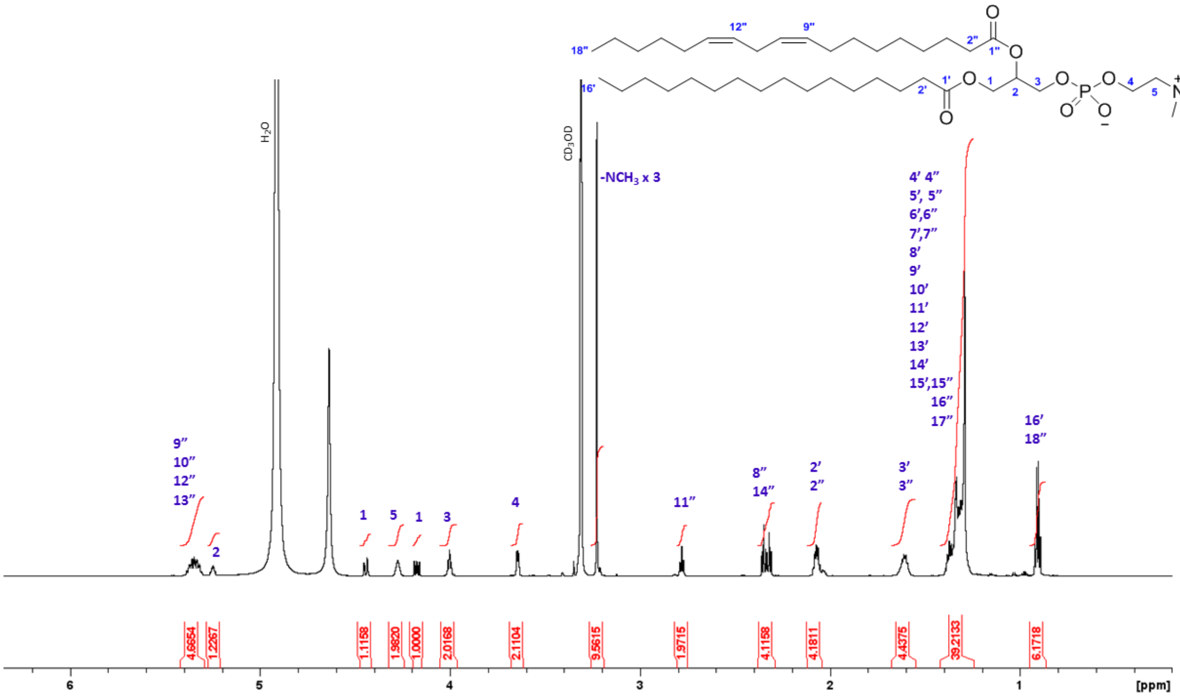


**(b)**

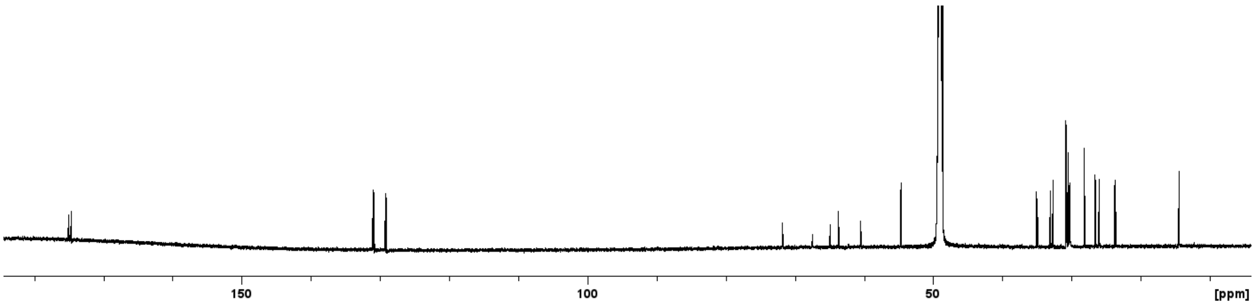


**Figure S8.** $^{1}H$- (a) and $^{13}C$-NMR spectrum of 1-palmitoyl-2-linoleoylphosphatidylcholine measured in $CD_3OD$. HRMS *m/z* 758.56768 [calcd for $C_{42}H_{81}NO_8P$ $(M + H)^+$ 758.56943].

(a)

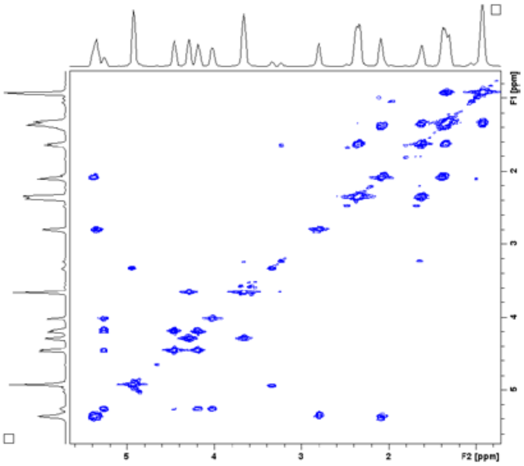

(b)

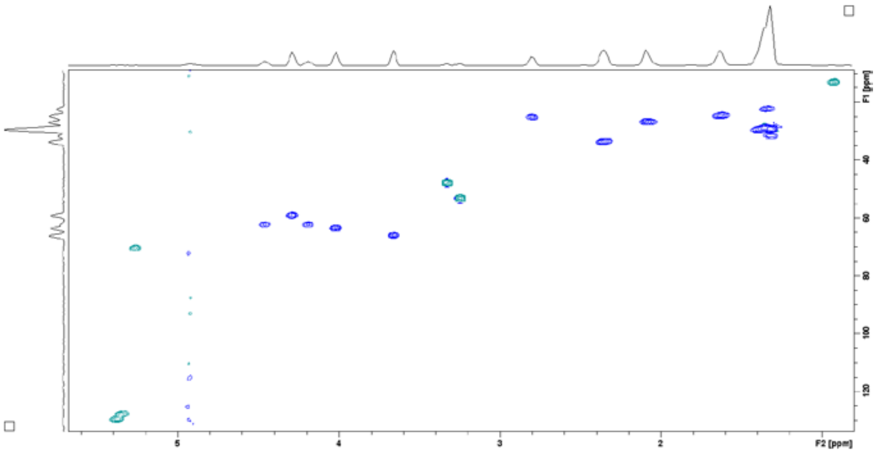

(c)

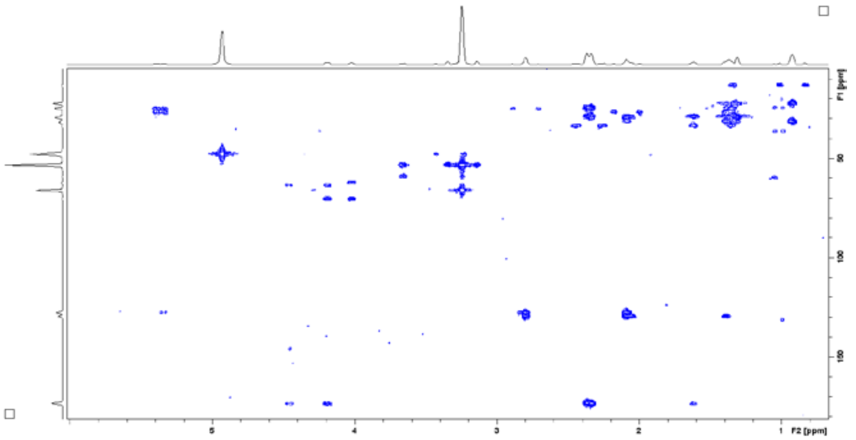

**Figure S9.** 2D-NMR spectra of 1- palmitoyl-2-linoleoylphosphatidylcholine COSY (a) HSQC (b) and HMBC (c) measured in $CD_3OD$.

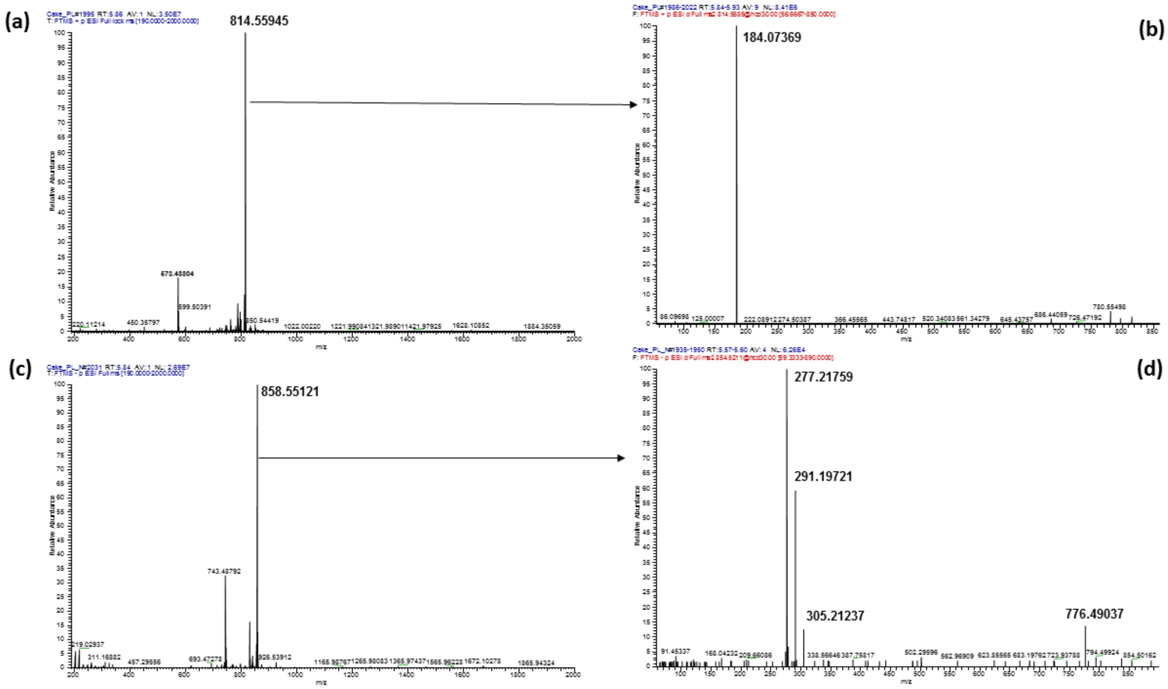


**Figure S10.** Mass spectrum of unknown phospholipid eluted around 6.00 min with molecular ion at *m/z* 814.55988 in positive mode (a) fragmentation ions of *m/z* 814.55988 in positive mode (b), mass spectrum of unknown phospholipid with molecular ion at m/z 858.55121 in negative mode (c) fragmentation ions of *m/z* 858.55121 (d).
